# Folate Status of Reproductive Age Women and Neural Tube Defect Risk: The Effect of Long-Term Folic Acid Supplementation at Doses of 140 µg and 400 µg per Day

**DOI:** 10.3390/nu3010049

**Published:** 2011-01-10

**Authors:** Nicola A. Hursthouse, Andrew R. Gray, Jody C. Miller, Meredith C. Rose, Lisa A. Houghton

**Affiliations:** 1 Department of Human Nutrition, University of Otago, Dunedin 9054, New Zealand; Email: hurni473@student.otago.ac.nz (N.A.H.); jody.miller@otago.ac.nz (J.C.M.); meredith.rose@otago.ac.nz (M.C.R.); 2 Department of Preventive and Social Medicine, University of Otago, Dunedin 9054, New Zealand; Email: andrew.gray@otago.ac.nz

**Keywords:** neural tube defects, blood folate status, folic acid fortification, supplementation

## Abstract

Primary prevention of most folate-responsive neural tube defects (NTDs) may not require 400 μg folic acid/day but may be achieved by attaining a high maternal folate status. Using RBC folate ≥906 nmol/L as a marker for NTD risk reduction, the study aimed to determine the change in blood folate concentrations in reproductive age women in response to long-term folic acid supplementation at 400 µg/day and 140 µg/day (dose designed to mimic the average daily folic acid intake received from New Zealand’s proposed mandatory bread fortification program). Participants were randomly assigned to a daily folic acid supplement of 140 µg (*n* = 49), 400 µg (*n* = 48) or placebo (*n* = 47) for 40 weeks. RBC folate concentrations were measured at baseline, and after 6, 12, 29 and 40 weeks. At 40 weeks, the overall prevalence of having a RBC folate <906 nmol/L decreased to 18% and 35% in the 400 µg and 140 µg groups, respectively, while remaining relatively unchanged at 58% in the placebo group. After 40 weeks, there was no evidence of a difference in RBC folate between the two treatment groups (*P* = 0.340), nor was there evidence of a difference in the odds of a RBC folate <906 nmol/L (*P* = 0.078). In conclusion, the average daily intake of folic acid received from the proposed fortification program would increase RBC folate concentrations in reproductive age women to levels associated with a low risk of NTDs.

## 1. Introduction

It is widely accepted that adequate maternal consumption of folic acid before pregnancy and during the early weeks of gestation can reduce the risk of having a child with a neural tube defect (NTD). As a result, public health authorities worldwide have recommended consuming 400 μg folic acid per day during the periconceptional period to reduce the risk of first occurrence NTDs [1,2,3,4]. This recommended dose is based mainly on the amount of folic acid observed to be associated with a reduction in NTDs in the majority of epidemiological studies [5,6,7,8,9]. It is also the level of intake that demonstrated a dramatic decline in NTD occurrence in a large-scale, prospective community intervention trial in China [10]. Whether an amount of folic acid lower than the current official recommendation would be efficacious is a critical question yet difficult to determine by way of randomized controlled trials. Alternatively, the relation of NTD risk to maternal red blood cell (RBC) folate concentrations may provide insight to the influence of varying levels of folic acid intake. In a case-control study carried out in Ireland, Daly *et al.* [11] documented a dose-response relationship between blood folate concentrations and the incidence of NTD, reporting that the lowest category of NTD risk occurred when maternal RBC folate concentrations were greater than 906 nmol/L. Achieving this protective folate concentration remains a challenge among reproductive age women.

Mandatory folic acid fortification of enriched cereal-grain products in the United States and Canada, implemented in 1998, has substantially improved the folate status of reproductive age women [12] and resulted in a significant decline in NTDs [13,14,15,16,17,18,19,20]. While implementation of fortification programs for the primary prevention of NTDs is still under debate in many countries, mandatory folic acid fortification of bread (140 µg folic acid/100 g) was gazetted in New Zealand for September 2009 but recently deferred until May 2012 [21]. At present, there are no national data on the folate status of New Zealand women, nor are there complete surveillance data on the national rate of NTDs. Using RBC folate as a marker of NTD risk, the aim of our study was to assess the percentage of reproductive age women who have RBC folate concentrations above the protective level, and to evaluate the long-term effect of 140 μg/day (dose designed to mimic the estimated average folic acid intake provided by the proposed bread fortification program in New Zealand) and 400 μg/day (dose recommended for primary prevention of NTDs) on blood folate status.

## 2. Experimental Section

### 2.1. Study Participants

A sample of healthy, reproductive age women (18–40 y) were recruited in July 2008 from the staff and student population at the University of Otago, Dunedin, New Zealand, and from the local community through advertisements in the newspaper and word-of-mouth. Women were not included in the study if they were pregnant, lactating, or were planning a pregnancy in the next 12 months. Additional exclusion criteria for the study were regular use of folic acid-containing supplements in the previous 6 months; self-reported history of cardiovascular, gastrointestinal, hepatic, renal or hematologic disease; and use of medications that interfere with folate metabolism (e.g., methotrexate, sulfasalazine, or anticonvulsants). Ethical approval was obtained from the Human Ethics Committee of the University of Otago, Dunedin, New Zealand, and all participants gave written, informed consent. This study was registered with the Australian New Zealand Clinical Trials Registry [22] as ACTRN12609000215224. 

### 2.2. Study Protocol

The study was a 40 week double-blinded, placebo-controlled trial conducted from August 2008 through May 2009. Participants attended an early morning clinic at baseline, and after 6, 12, 29 and 40 weeks. At the baseline visit, sociodemographic and general health data were collected using a self‑administered questionnaire; height and weight were taken according to standardized procedures [23] and measured to the nearest 0.1 cm and 0.1 kg, respectively. At the completion of the baseline visit, participants were provided with electronic digital scales accurate to 1 g (Model 1017; Salter Housewares Ltd., Tonbridge, England) and received oral and written instructions on how to weigh and record all food and beverages consumed over an assigned 3-day period. Participants were then randomly assigned through the use of non-identifying group codes to receive either 400 μg folic acid/day, 140 μg folic acid/day, or placebo. At each clinic visit including baseline, fasting blood samples (15 mL collected ≥12 h after an overnight fast) were obtained by venipuncture before the study tablet was administered. 

### 2.3. Supplements

The study supplements were manufactured by New Zealand Nutritionals Ltd (Christchurch, New Zealand) as hard tablets, each containing microcrystalline cellulose as a filler (placebo), and either 140 μg (317 nmol) folic acid, or 400 μg (907 nmol) folic acid. Folate contents of the tablets were analyzed by microbiological assay in our laboratory at the beginning of the study. The actual amounts in the tablets aimed to provide 140 μg and 400 μg folic acid were 133 μg and 359 μg folic acid, respectively. Participants, investigators and biostatistician were blinded to the treatment. Supplements were coded by a third-party and randomization data was kept strictly confidential in a sealed envelope until all of the data analyses had been performed. Participants were instructed to take one tablet per day and to return all unused pills. Three bottles of tablets were prepared for each participant, and distributed at baseline, and weeks 12 and 29. Compliance with respect to supplement intake was assessed by counting returned pills. 

### 2.4. Dietary Folate Assessment

Dietary intake of folate was determined via 3-day weighed food records completed at baseline. The diet records were completed over two non-consecutive weekdays and one weekend day. The total folate intakes, in µg, were tabulated using an electronic version of the 2006 New Zealand Food Composition Tables [24] and expressed as an overall mean of the 3-day intake. Any unavailable foods in the composition table were substituted with an item of the same type with a similar folate level or, if available, estimated from the amount stated on the manufacturer label. In total, 128 3-day baseline diet records were entered and analysed. Dietary folate intakes may have varied over the 40 week intervention period, particular due to seasonal fluctuations in price and availability of folate-rich foods. 

### 2.5. Blood Sampling and Laboratory Analysis

Blood samples were collected in EDTA-treated tubes, transported on ice and processed within one hour of collection. After measurement of the hematocrit, red blood cell hemolysates were prepared (dilution (1:10) of whole blood with 1% ascorbic acid) and incubated at 37 °C for 30 min before storage at −80 °C. The remaining whole blood was centrifuged (1500 g for 15 min at 4 °C) to separate the plasma and aliquots were stored immediately at −80 °C. Plasma and whole blood folate concentration was determined by microbiological assay [25] using test organism chloramphenicol-resistant *Lactobacillus rhamnosus* (ATCC 27773; American Type Culture Collection, Manassas, VA). Erythrocyte folate concentration was calculated from whole-blood values using individual packed cell volumes and after correction for plasma folate concentration. To avoid between-run variation, all study visit samples from each participant were measured in one batch. Accuracy and inter-assay variability were monitored with the use of two external quality controls: whole blood folate standard (National Institute for Biological Standards and Control, Hertfordshire, United Kingdom) with a certified value of 29.5 nmol/L (mean 27.3 (SD 4.6); CV 17%) and a human serum folate standard reference material (SRM 1955, National Institute of Standards and Technology, Gaithersburg, U.S.) with a total folate information value as determined by microbiological assay of 44 nmol/L (mean 42.5 (SD 8.7); CV 20%). A plasma folate concentration <6.8 nmol/L or a RBC folate concentration <317 nmol/L was used to indicate folate deficiency [26]. 

### 2.6. Statistical Analysis

All statistical analyses were performed by using Stata (version 11, Stata Corp, College Station, TX). Characteristics of participants completing the study and dropping out before the final blood sample were compared using t-tests for continuous variables (after log-transformations where appropriate) and χ^2^ tests for categorical variables (with Fisher’s exact test used where expected cell counts were below 5 for more than 20% of cells). The outcome variables were plasma and RBC folate concentrations and suboptimal RBC concentration (determined as <906 nmol/L). The following known or potential predictors were identified a priori: age, BMI, education, ethnicity, use of oral contraceptives and dietary folate. Education was collapsed into three categories: secondary school or less, post-secondary education (university or college), or advanced degree. Participant ethnicity was classified as New Zealand European, Maori and Pacific Peoples, Asian, or other ethnicities. Plasma and RBC folate concentrations were natural log transformed where this resolved issues with skew and/or heteroscedasticity in residuals. Linear and logistic mixed models were used to assess intervention group effects and included a random participant effect. Difference in changes between groups was assessed using treatment group-by-time interactions. Selected error covariance structures (autoregressive, toeplitz, and unstructured) were investigated for linear mixed models to see if they improved model fit according to the Akaike information criterion (AIC). Non-linear associations were investigated, and where appropriate, modelled using fractional polynomials. Variables with *P* < 0.25 in models including treatment group, time, and their interaction were included in the final regression models. All tests were 2-sided with statistical significance as determined by *P* < 0.05.

## 3. Results

**Figure 1:**
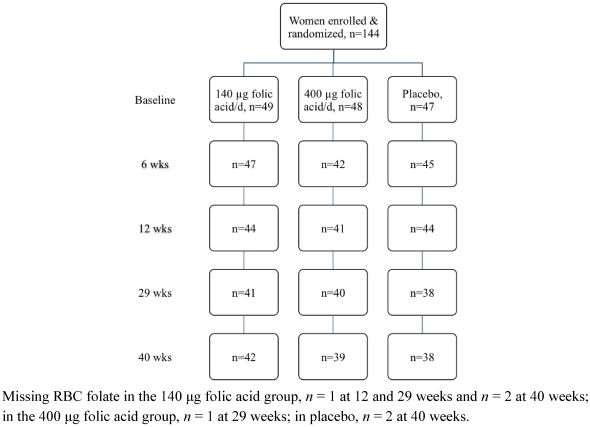
Flow diagram of the women who participated in the randomised trial.

Of the 144 women enrolled in the study, 119 completed the intervention trial (Figure 1). Baseline characteristics of the study population by treatment group are presented in Table 1. The majority of participants were university educated, and identified as New Zealand European. Nearly one-third of the participants were classified as overweight or obese. Overall folate status of the study population was adequate (Table 1). Twenty-five participants were lost to the study over the 40 week intervention period with one participant conveying her intent to conceive and the remainder citing lack of time or personal reasons. No statistically significant evidence of differences were observed in treatment group, participant age, BMI category, ethnicity, education or baseline plasma and RBC folate concentrations between those participants who remained in the study and those who withdrew before the end of the study (all *P* ≥ 0.325). With respect to study supplement intake, 93% (134 out of 144) participants returned at least one of their three study bottles and of these, 90% (120 out of 134) had compliance of 70% or higher. 

**Table 1:** Baseline characteristics of study population ^1^.

	All (*n* = 144)	Placebo (*n* = 47)	140 µg folic acid/day (*n* = 49)	400 µg folic acid/day (*n* = 48)
Age (y) ^2^	24.5 ± 5.9	24.5 ± 5.6	24.2 ± 6.2	24.9 ± 5.8
Weight (kg) ^2^	65.5 ± 12.3	65.5 ± 12.2	66.5 ± 12.1	64.5 ± 12.7
BMI (kg/m²) ^2^	23.7 ± 3.9	23.6 ± 3.8	24.2 ± 4.0	23.4 ± 4.0
BMI Category, *n* (%)				
<18.5	7 (5)	2 (4)	2 (4)	3 (6)
18.5–24.9	96 (67)	32 (68)	31 (63)	33 (69)
25.0–29.9	32 (22)	10 (21)	12 (25)	10 (21)
≥30.0	9 (6)	3 (6)	4 (8)	2 (4)
Ethnicity, *n* (%) ^3^				
New Zealand European	109 (76)	35 (75)	35 (71)	39 (81)
Maori and Pacific Peoples	7 (5)	4 (9)	2 (4)	1 (2)
Asian	17 (12)	6 (13)	8 (16)	3 (6)
Other ethnicities	11 (8)	2 (4)	4 (8)	5 (10)
Education, *n* (%) ^3^				
Secondary school or less	17 (12)	3 (6)	9 (18)	5 (10)
Post-secondary education	105 (73)	39 (83)	31 (63)	35 (73)
Advanced degree	22 (15)	5 (11)	9 (18)	8 (17)
Oral contraceptive use, *n* (%)	63 (44)	22 (47)	20 (41)	21 (44)
Dietary Folate (µg/day) ^4,5^	255.0 ± 1.5	246.7 ± 1.5	248.0 ± 1.6	271.5 ± 1.5
Plasma folate (nmol/L) ^4^	19.9 ± 1.8	21.4 ± 1.6	19.2 ± 1.8	19.3 ± 1.9
<6.8 nmol/L, *n* (%)	2 (1)	0 (0)	2 (1)	0 (0)
Red blood cell folate (nmol/L) ^4^	753.2 ± 1.6	808.5 ± 1.5	700.4 ± 1.6	756.8 ± 1.6
<317 nmol/L, *n* (%)	4 (3)	1 (2)	1 (2)	2 (4)
<906 nmol/L, *n* (%)	95 (66)	30 (64)	33 (67)	32 (67)

^1^ There were no significant differences between the three groups; ^2^ Mean ± SD; ^3^ May not be equal to 100% due to rounding; ^4^ Geometric mean ± geometric SD; ^5^ Dietary folate intake from food sources only including natural folate and folic acid from fortified foods (due to missing data, *n* = 128 (*n* = 43 placebo, *n* = 43, 140 μg/day, and *n* = 42, 400 μg/day)).

The geometric mean plasma and RBC folate concentrations at baseline and over the 40 week intervention period are shown in Table 2. Error covariance structures did not improve model fit as measured by AIC, and mixed models included only random effects for participants to account for repeated measures. For both plasma and RBC folate, statistically significant interactions between time and intervention were observed (both *P* < 0.001). Relative to the placebo group, plasma folate increased statistically significantly in both supplemented groups; furthermore, the mean (95% CI) increase in plasma folate of 107% (63, 164) in the 400 μg group was statistically significantly greater (*P* = 0.007) than the group receiving 140 μg folic acid/day (50% (18, 90)). In contrast, after 40 weeks of supplementation, there were no statistically significant differences in the observed increases in RBC folate concentrations between the two treatment groups (63% (95% CI: 39, 92) *versus* 51% (29, 78), *P* = 0.340).

**Table 2:** Plasma and red blood cell folate concentrations at baseline and over the 40 week intervention period ^1^.

Measurement and treatment group	Baseline	Week 6	Week 12	Week 29	Week 40	Difference in change from baseline to 40 weeks (%) 2
**Plasma folate (nmol/L)**						
Placebo	21.1 (17.8, 24.4)	20.3 (17.1, 23.5)	19.7 (16.6, 22.9)	20.6 (17.2, 24.1)	21.0 (17.4, 24.5)	–
140 μg folic acid/day	19.5 (16.5, 22.5)	26.6 (22.5, 30.8)	28.0 (23.6, 32.5)	31.0 (25.9, 36.0)	29.0 (24.3, 33.7)	49.8 (18.1, 90.1) ^3^
400 μg folic acid/day	19.2 (16.3, 22.2)	37.3 (31.2, 43.3)	38.3 (32.1, 44.6)	37.3 (31.1, 43.4)	39.5 (33.0, 46.1)	107.0 (62.7, 163.8) ^4,5^
**RBC folate (nmol/L)**						
Placebo	794.4 (696.7, 892.1)	763.8 (668.8, 858.8)	707.8 (619.2, 796.5)	729.0 (634.3, 823.7)	819.0 (711.1, 926.8)	–
140 μg folic acid/day	711.8 (626.1, 797.5)	834.5 (732.8, 936.1)	849.0 (743.4, 954.7)	987.0 (861.8, 1112.2)	1111.3 (970.3, 1252.3)	51.4 (29.1, 77.6) ^4^
400 μg folic acid/day	755.5 (664.2, 846.8)	910.8 (796.2, 1025.3)	940.1 (821.2, 1059.1)	1121.7 (977.9, 1265.4)	1273.4 (1110.2, 1436.6)	63.4 (39.2, 92.0) ^4^

^1^ Adjusted geometric means (95% CI) from models controlling for age, BMI and education (plasma folate) and education only (RBC folate). There were statistically significant time × treatment interactions for changes in plasma and RBC folate, *P* < 0.001 in both cases; ^2^ Relative to the placebo group; ^3^ Statistically significantly different change from baseline compared to placebo, *P* = 0.001; ^4^ *P* < 0.001; ^5^ Statistically significantly different change from baseline compared to 140 µg folic acid/day, *P* = 0.007.

**Figure 2:**
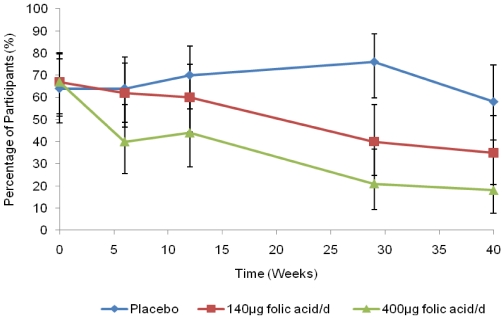
Unadjusted percentage of red blood cell folate concentrations <906 nmol/L (95% CI).

The decreasing prevalence of RBC folate concentration <906 nmol/L in the supplemented groups is illustrated in Figure 2. From baseline to 40 weeks, the prevalence of RBC folate <906 nmol/L decreased nearly 3-fold in the 400 μg group, from 67% to 18%, and approximately 2-fold in the 140 μg group (67% to 35%). The prevalence in the placebo group was relatively unchanged (64% to 58%). From week 6, the 400 µg treatment group had statistically significantly decreased odds of having a RBC folate concentration <906 nmol/L compared with those in the placebo group after adjusting for education and the repeated measures (*P* = 0.009). There was no evidence that the odds changed among women taking 140 µg until week 29 (*P* < 0.001). At week 40, no statistically significant differences were detected in the odds ratio of having a RBC folate <906 nmol/L between treatment groups (*P* = 0.078).

## 4. Discussion and Conclusions

The present study demonstrates that although the prevalence of folate deficiency is low, nearly two‑thirds of our study population had baseline RBC folate concentrations below the level thought to be protective against NTDs (*i.e.*, ≥906 nmol/L). Since 1996, New Zealand has permitted voluntary folic acid fortification of selected foods, however few foods have been fortified under this policy. It is estimated that the mandatory fortification of bread will increase average daily folic acid intakes among the target population by 136 μg. Our intervention trial was designed to evaluate changes in RBC folate at the proposed estimated daily intake level compared to the current official recommended dose of 400 μg folic acid/day for the primary prevention of NTD. After 40 weeks supplementation, blood folate status increased significantly in both treatment groups, however there was no evidence that mean RBC folate concentrations were significantly higher in the group of women taking 400 μg folic acid/day as compared to the 140 μg group. Moreover, there was no evidence of a difference in the odds of having a RBC folate <906 nmol/L between treatment groups at 40 weeks. 

To our knowledge, this is the longest intervention trial conducted to evaluate changes in blood folate indices to moderate folic acid intake levels. Previous intervention studies [27,28,29] have documented that RBC folate concentrations do not appear to reach a plateau with daily folic acid intakes between 100–400 μg after 24 weeks. Our data support and extend these findings, as demonstrated by a continuous increase in RBC folate concentration in both folic acid treatment groups over the 40 week intervention period. In our study, mean RBC folate levels increased by 275 nmol/L among study participants receiving 140 μg folic acid/day over 29 weeks—a finding similar to that found by Venn *et al.* [27] in which RBC folate increased by 251–275 nmol/L after 24 weeks of low dose folate supplementation (≈100 μg folic acid/day). From 29 to 40 weeks, we observed a further increase in mean RBC folate concentrations of 124–151 nmol/L in both folic acid regimens. However, unlike the study by Venn *et al.* [27], we did not measure changes in plasma homocysteine. Several epidemiologic studies have demonstrated a positive association between homocysteine and risk of NTDs [30]. Venn *et al.* [27] reported a 9% reduction in plasma homocysteine concentrations following supplementation with 100 μg folic acid/day over 24-week period. More recently, a 6-month supplementation study of 200 μg folic acid/day showed that this intake level was as effective as ≥400 μg folic acid/day in lowering homocysteine concentrations in a group of healthy, older adult participants [31]. 

Recent estimates in the United States and Canada show that folic acid fortification of the food supply has provided an additional intake of about 100 to 150 μg/day to reproductive age women [32,33]. Measurement of blood folate from the third National Health and Nutrition Examination Survey (NHANES III, 1998–1994), representing the period before fortification, through to three 2-y post-fortification survey periods covering 1999–2004 show that RBC folate concentrations increased by approximately 50% in every age group after the introduction of folic acid fortification [12]. This increase in blood folate compares favourably to our observed increase of 51% in mean RBC folate concentration after ingestion of 140 μg folic acid/day over 40 weeks. Furthermore, the proportion of study participants in the 140 μg/day group having a RBC folate concentration ≥906 nmol/L (65%) is also consistent with the 60–70% of reproductive age Canadian women reported to have attained this optimal level of blood folate after mandatory folate fortification of the food supply [34,35]. 

Folic acid fortification in countries that have implemented mandatory policies has proven to be one of the most successful public health interventions in reducing the prevalence rate of NTDs. In Canada, De Wals *et al.* [15] indicated that the birth prevalence of NTDs decreased from 1.58 per 1000 births before fortification to 0.86 per 1000 births post-fortification across seven Canadian provinces. This prevalence rate is consistent with a recent systematic review, which estimated that approximately 7–8 NTD cases per 10,000 births were still observed (when measuring the NTD prevalence at birth or from abortion) after the implementation of fortification or following periconceptional supplement use in primary prevention studies [36]. Moreover, recent data from the US National Birth Defects Prevention Study (1998–2003) reported that periconceptional use of folic acid supplements in the post-fortification era no longer appears to further reduce NTD risk [37] suggesting that fortification may have provided the necessary level of folic acid needed to prevent most folate-responsive NTDs [37,38]. 

The overall prevalence of NTDs in New Zealand is difficult to determine accurately due to inadequate data collection around the number of NTD-affected pregnancies. Complete data (terminations, stillbirths and live births) are only available for 1998 to 2003. These annual totals demonstrate a downward trend in the rate of NTD affected pregnancies rate from 17.4 to 11.2 per 10,000 births [39]. In 2003, NTD rates were the highest in early pregnancy terminations (5.7 cases per 10,000 total births), followed by live births (3.4 per 10,000) and foetal deaths (2.1 per 10,000) [40]. An assessment of the potential effect of incremental increases in folic acid intake on NTDs in New Zealand was undertaken in 2005 by Bower *et al.* [41]. On the basis of this assessment, the proposed mandatory bread fortification program is anticipated to reduce the prevalence of NTDs by 4–14% (or up to 20%) per year from an estimated 70–75 NTD-affected pregnancies. This projection was calculated using the dose-response relationship in serum folate described by Wald *et al.* [42] and the proportional risk of NTDs to a given serum folate level from nested case-control analysis by Daly *et al.* [11]. It has been noted, however, that the model developed by Wald *et al.* [42] likely underestimates the rise in serum folate concentrations (2.3 nmol/L increase for every 100 μg intake of folic acid/day) because 4 of the 6 studies included in the analysis were not of sufficient duration to achieve a plateau in serum folate concentrations (*i.e.*, ~6 weeks needed when ≤200 μg folic acid/day is consumed) [43]. Thus, the projected NTD reduction attributed to the proposed bread fortification is likely to be underestimated. Our results support this notion as reflected by an observed rise of 8.5 nmol/L in mean plasma folate concentrations at 12 weeks among participants receiving 140 μg folic acid/day compared to the expected rise of 3.2 nmol/L derived using the Wald model [42]. Furthermore, the change in steady state plasma folate concentration among our women consuming 140 μg folic acid per day is highly comparable to the calculated rise of 8.2 nmol/L derived from a regression model by Quinlivan and Gregory [44], which included data from 11 intervention studies of sufficient duration. 

Mandatory folic acid fortification of bread in New Zealand has been deferred until May 2012 due to public concerns regarding cost-effectiveness and the impact on consumer choice [21]. There are also a large range of public concerns over mandatory fortification in New Zealand and the effects it may have on non-target population groups. To date, there are no known safety concerns at the low-to-moderate level of intake resulting from fortification of the food supply with folic acid [45]. Currently, New Zealand recommends that women planning a pregnancy take 800 μg folic acid daily for 4 weeks prior to conception, and 12 weeks after to reduce the risk of NTDs [46]. Results from a small number of surveys conducted to assess supplement use among reproductive age women suggest that only a minority of women comply with the recommendation [47,48,49]. While optimal folic acid strategies continue to be debated worldwide, the findings of this trial are important as they allow for the evaluation of the impact of the proposed folic acid fortification programme on the target population. Using the endpoint of blood folate, our results suggest that the level of folic acid provided by the fortification program would increase RBC folate concentrations to a level associated with a low risk of NTDs. Moreover, given the greater response of plasma folate concentration to the proposed estimated level of intake, NTD risk reduction, as currently modeled, is likely underestimated. This is of particular importance in light of recent speculations that a similar increase in folic acid intake (provided by the U.S fortification program) may result in the prevention of most folate-related NTDs. 
